# Supplementary figures and images for: Crustacean Mab21 proteins drive tissue-specific antiviral immunity by activating IKKε outside the canonical nucleic-acid sensing paradigm
Source: PLoS Pathog. 2026 Feb 17;22(2):e1013986. doi: 10.1371/journal.ppat.1013986 (PMC12928593; doi:10.1371/journal.ppat.1013986)

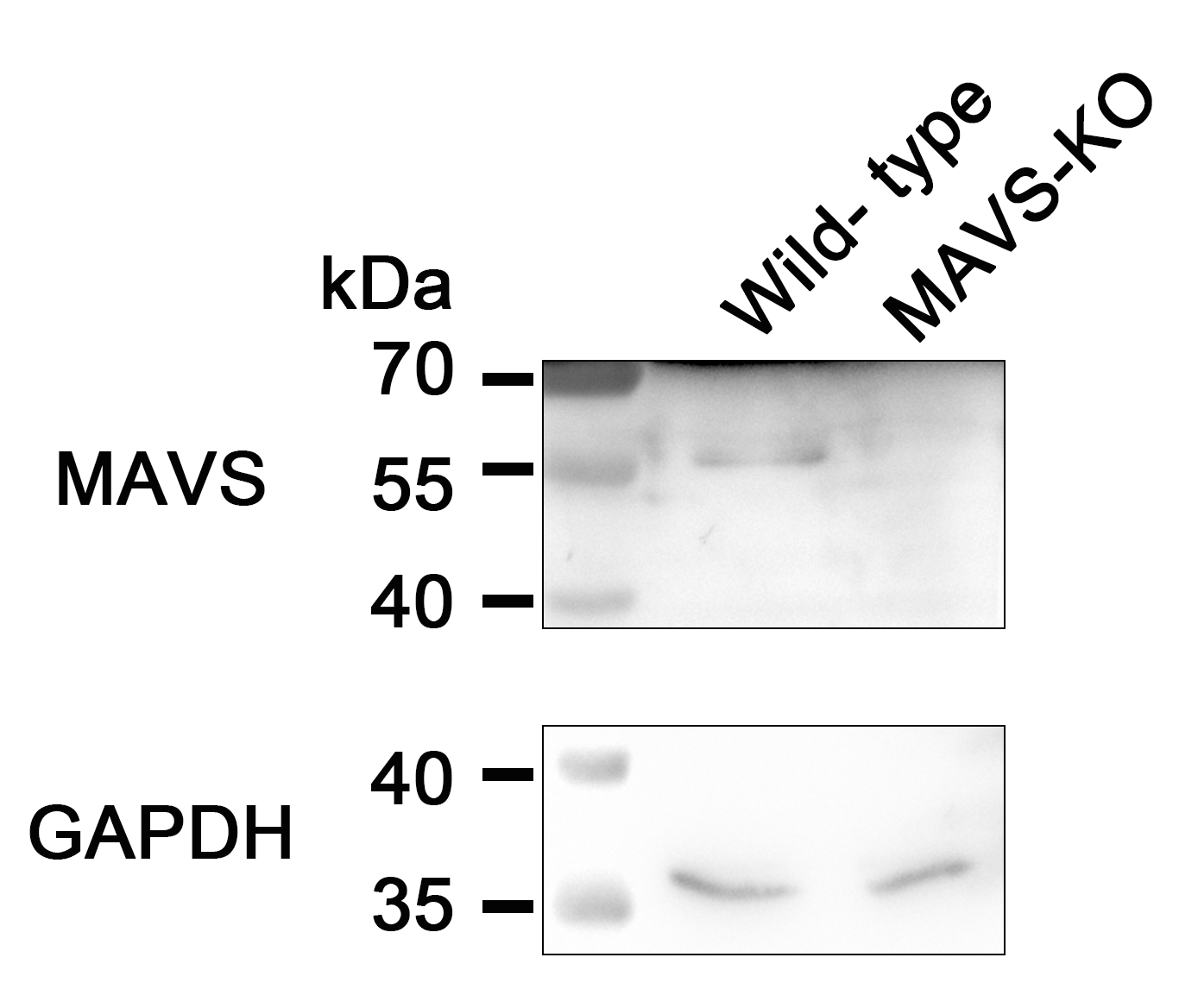

Supplement: S1 Fig — (TIF) [file ppat.1013986.s001.tif]

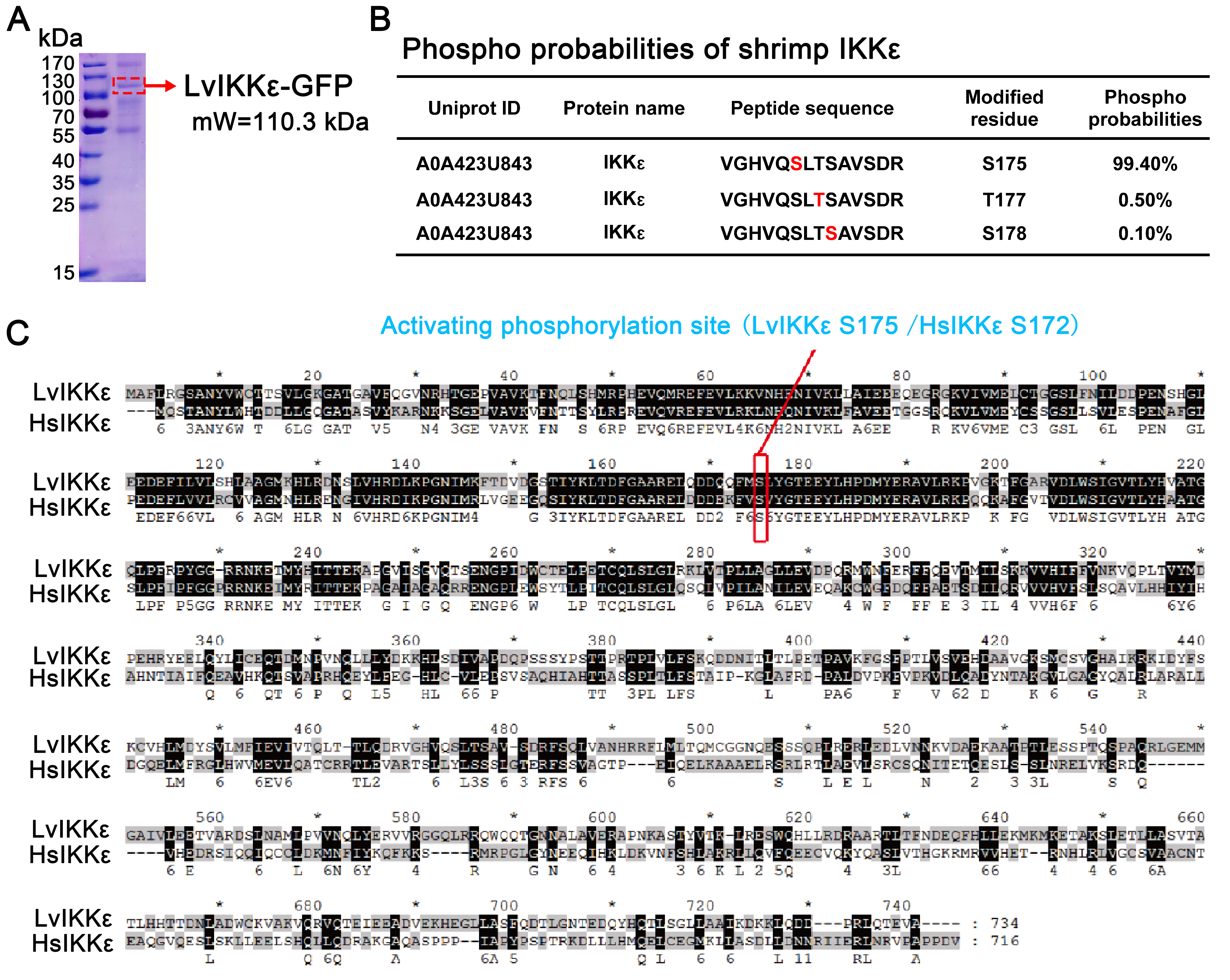

Supplement: S2 Fig — (A) IP assay of GFP tagged LvIKKε in HepG2 cells under poly(I:C) stimulation. The protein band corresponding to the eluted products via anti-GFP Magnetic Beads was analyzed by liquid chromatography-tadem mass spectrometry (LC-MS/MS). (B) Phosphorylation probabilities of LvIKKε analyzed by LC-MS/MS. (C) Multiple sequence alignment of LvIKKε and HsIKKε. (TIF) [file ppat.1013986.s002.tif]

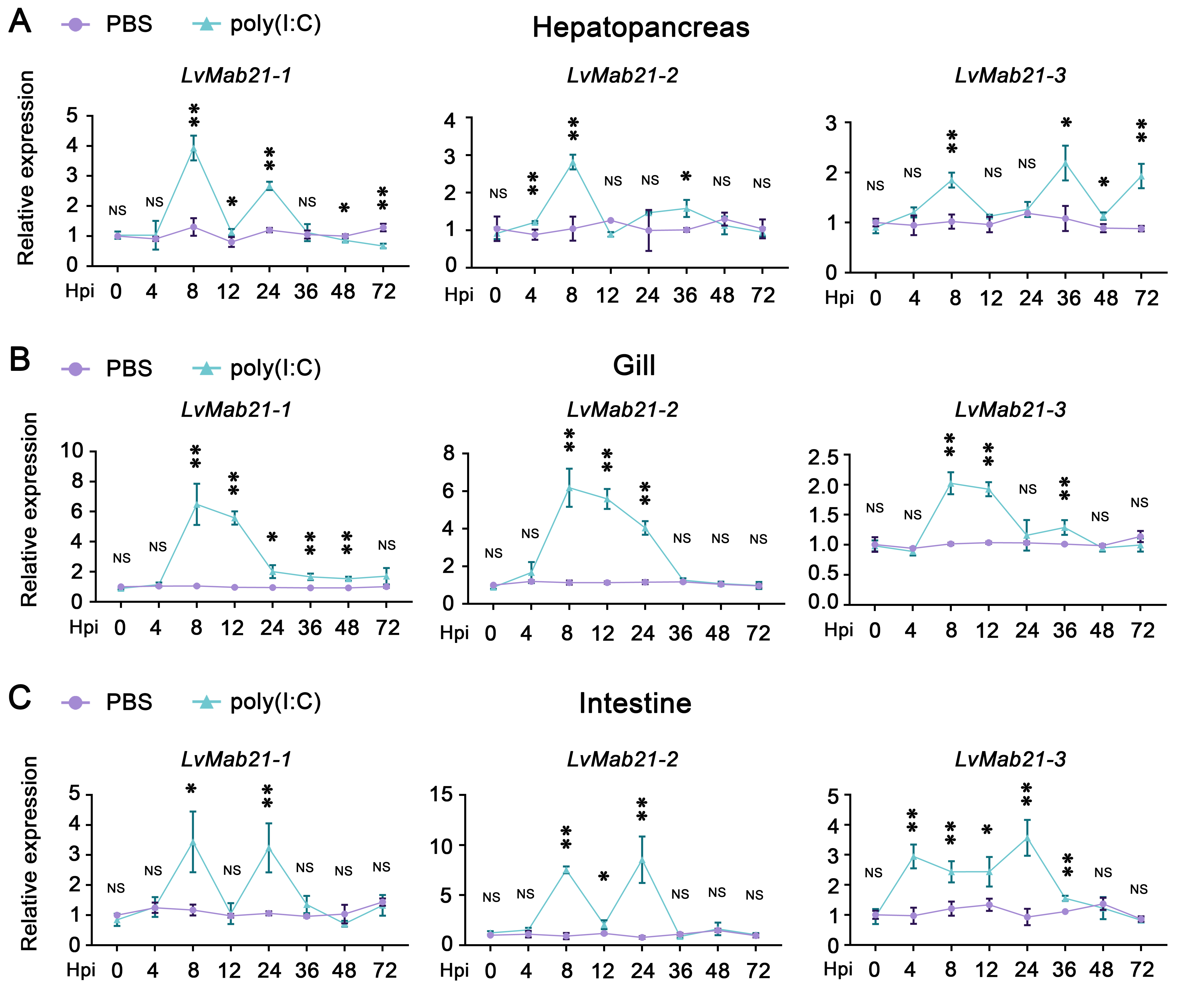

Supplement: S3 Fig — (TIF) [file ppat.1013986.s003.tif]

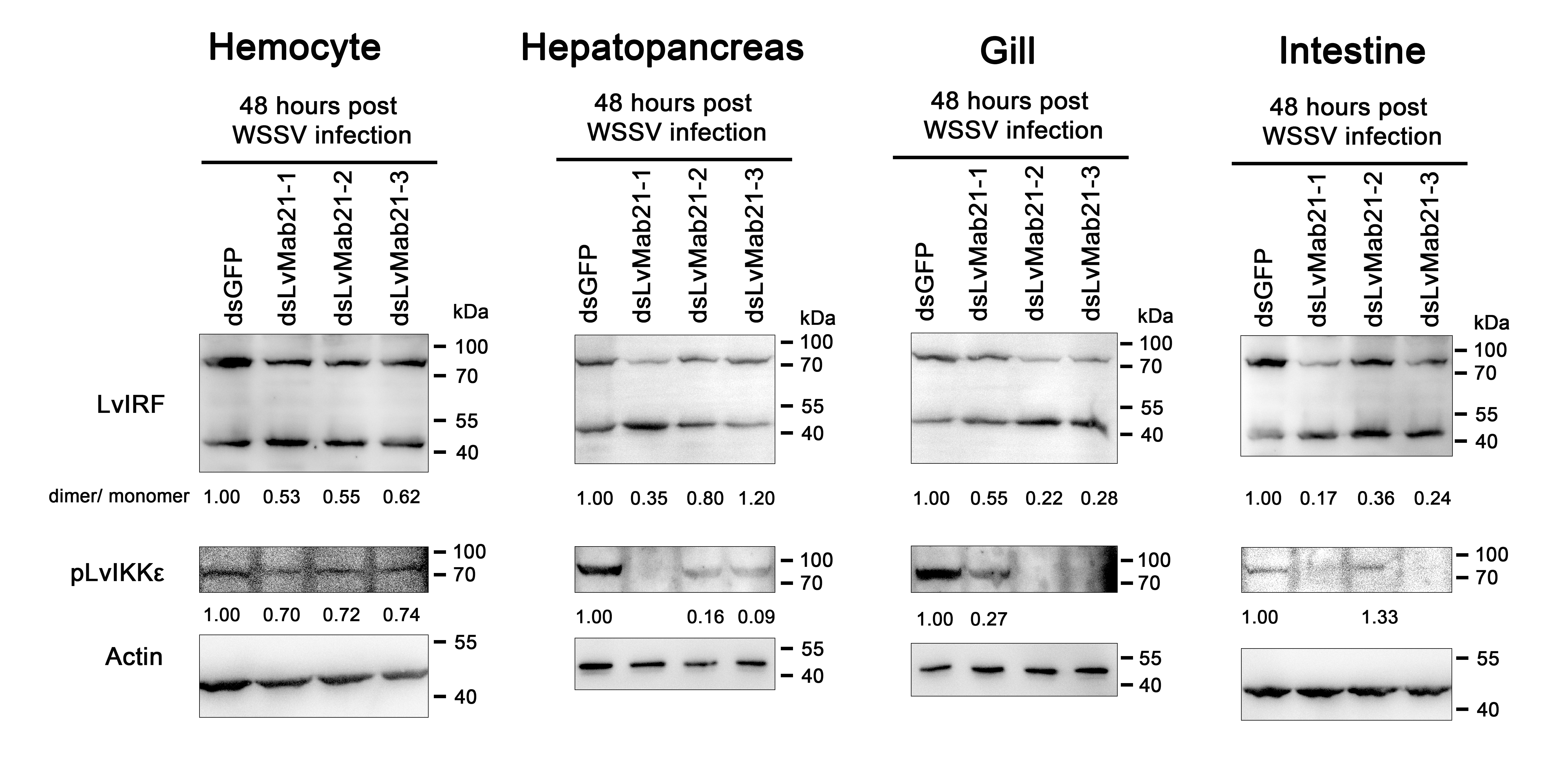

Supplement: S4 Fig — (TIF) [file ppat.1013986.s004.tif]
