# Supplementary material for: Crustacean Mab21 proteins drive tissue-specific antiviral immunity by activating IKKε outside the canonical nucleic-acid sensing paradigm
Source: PLoS Pathog. 2026 Feb 17;22(2):e1013986. doi: 10.1371/journal.ppat.1013986 (PMC12928593; doi:10.1371/journal.ppat.1013986)
Supplement: S1 Table — (DOCX) [file ppat.1013986.s005.docx]

**S1 Table. Primers used in this study.**

| Primers | Sequences (5’ to 3’) |
| --- | --- |
| Primers for dsRNA synthesis | |
| LvMab21-1-F | ATGAACCCGAACATGGACCGCCTCAA |
| LvMab21-1-R | CAGGGTATCGAAATCCTCTTCAAAGCAC |
| LvMab21-1-T7-F | TAATACGACTCACTATAGGATGAACCCGAACATGGACCGCCTCAA |
| LvMab21-1-T7-R | TAATACGACTCACTATAGGCAGGGTATCGAAATCCTCTTCAAAGCAC |
| LvMab21-1-F | TTCAACTTCGTGGACGACGGCAGCAT |
| LvMab21-1-R | CAGCAGATCGAATCCTTCTGTCTTGACCTC |
| LvMab21-1-T7-F | TAATACGACTCACTATAGGTTCAACTTCGTGGACGACGGCAGCAT |
| LvMab21-1-T7-R | TAATACGACTCACTATAGGCAGCAGATCGAATCCTTCTGTCTTGACCTC |
| LvMab21-1-F | CGGCGGTCGGTGGGAGGGTCTC |
| LvMab21-1-R | TCGGCCAGATGCCATAACACTTGA |
| LvMab21-1-T7-F | TAATACGACTCACTATAGGCGGCGGTCGGTGGGAGGGTCTC |
| LvMab21-1-T7-R | TAATACGACTCACTATAGGTCGGCCAGATGCCATAACACTTGA |
| GFP-F | CGACGTAAACGGCCACAAGTT |
| GFP-R | ATGGGGGTGTTCTGCTGGTAG |
| GFP-T7-F | GGATCCTAATACGACTCACTATAGGCGACGTAAACGGCCACAAGTT |
| GFP-T7-R | GGATCCTAATACGACTCACTATAGGATGGGGGTGTTCTGCTGGTAG |
| Primers for quantitative reverse transcription PCR (qRT-PCR) | |
| *HsIFN1-β*-F | GCTTGGATTCCTACAAAGAAGCA |
| *HsIFN1-β*-R | ATAGATGGTCAATGCGGCGTC |
| *Hsβ-actin*-F | ACGGCATCGTCACCAACTG |
| *Hsβ-actin*-R | GAGCCACACGCAGCTCATT |
| *LvMab21-1*-F | AAATGCCCGCGATCCTTGCC |
| *LvMab21-1*-R | CCCGTGTTGACGACTTCGTTGAT |
| *LvMab21-2*-F | GTACGAATGCGAGAAGCACCCTCGGG |
| *LvMab21-2*-R | CAGGAGGTTGATTGACGGGAGGAAGTAGTG |
| *LvMab21-3*-F | TCGGGGACCGCATCAATGGC |
| *LvMab21-3*-R | GGTACTCAGTTCTGGTGAGCAGGT |
| *LvVago4*-F | ACGACGAGTTCACGAATTGGATC |
| *LvVago4*-R | ACGGCATCTTACCTCAAGAGTC |
| *EF1α*-F | GTATTGGAACAGTGCCCGTG |
| *EF1α*-R | ACCAGGGACAGCCTCAGTAAG |
| Primers of wsv069 (IE1) for absolute quantitative PCR | |
| WSSV-F | TGTTTTCTGTATGTAATGCGTGTAGGT |
| WSSV-R | CCCACTCCATGGCCTTCA |
| TaqMan probe-WSSV | CAAGTACCCAGGCCCAGTGTCATACGTT |
| Primers for pGL3 construction | |
| pGL3-LvVago4-F | AATGGTACCTCTGGAGTGCGGGCGTG |
| pGL3-LvVago4-R | TTCAGATCTCACAGGACCAGGGCGACTT |
| Primers for protein expression | |
| LvSTING-F | AGGGGTACCATGAAGGGAGACGAGCTGGTC |
| LvSTING-R | AACGGGCCCGCAAAACAAAAGAGATTCTGCCGCT |
| LvLvIRF-F | CGGGTACCATGCCGCCATCTTTCACCAATGTCC |
| LvLvIRF-R | GCTCTAGACGGCAACGTCCTCTCGCCGGCATAC |
| LvLvIKKε-F | CGGGTACCATGGCATTTCTGCGAGGATC |
| LvLvIKKε-R | TGGGGCCCCGCAACCTCAGTTTGTAATCTTG |
| LvLvIKKε-S175A-F | CAGCAATTCATGGCTCTCTATGGAA |
| LvLvIKKε-S175A -R | TTCCATAGAGAGCCATGAATTGCTG |
| HsSTING-F | CCGGAATTCATGCCCCACTCCAGCCTG |
| HsSTING-R | CCGCTCGAGAGAGAAATCCGTGCGGAGAG |
| LvMab21-1-F | AGGGGTACCATGAACCCGAACATGGACCGCCTCA |
| LvMab21-1-R | AACGGGCCCTTAGAGGTCCGTCGACGCCTCCTCC |
| LvMab21-2-F | AGGGGTACCATGGGCGGCGCGCGGCCCTTTG |
| LvMab21-2-R | AACGGGCCCTCACAGCTTCTCCAGGCTCTTGGAG |
| LvMab21-3-F | AGGGGTACCATGTTGGTGCCCAGTGAAGGTGTCT |
| LvMab21-3-R | AACGGGCCCTTAACACTGCTGAAGGTACTCAGTT |
| HscGAS-F | CGGAATTCATGCAGCCTTGGCACGGAAAGGC |
| HscGAS-R | ATTTGCGGCCGCTCAAAATTCATCAAAAACTGGAAAC |
| DmcGLR1-F | GGGGTACCATGGCGATGAATTTGGAAAATATCG |
| DmcGLR1-R | CCCTCGAGTTACAATTTGTATGTGGTGTTCTTACTAAAAG |
