# Supplementary material for: Crustacean Mab21 proteins drive tissue-specific antiviral immunity by activating IKKε outside the canonical nucleic-acid sensing paradigm
Source: PLoS Pathog. 2026 Feb 17;22(2):e1013986. doi: 10.1371/journal.ppat.1013986 (PMC12928593; doi:10.1371/journal.ppat.1013986)
Supplement: S2 Table — (DOCX) [file ppat.1013986.s006.docx]

**S2 Table. Proteomic identification of IP products based on LC-MS/MS analysis.**

| **Uniprot ID** | **Protein name** | **Unique peptide count** | **Cover percent** | **​Isoelectric Point** |
| --- | --- | --- | --- | --- |
| **/** | **IKKε-GFP** | **268** | **54.4%** | **5.9** |
| **A0A423U843** | **IKKε** | **204** | **49.4%** | **5.85** |
| **A0A3R7PMY7** | **Putative neuroblastoma-amplified sequence** | **87** | **0.5%** | **4.89** |
| **A0A3R7LZ58** | **DNA topoisomerase I** | **12** | **7.5%** | **6.03** |
| **A0A3R7MSV7** | **RNA helicase** | **5** | **7.9%** | **9.11** |
| **A0A423SYA2** | **Actin 1** | **5** | **16.8%** | **5.24** |
| **A0A3R7N7T1** | **26S proteasome non-ATPase regulatory subunit 2** | **4** | **4.3%** | **5.1** |
| **A0A423U7J2** | **Ubiquitin-activating enzyme E1** | **2** | **1.2%** | **5.15** |
| **A0A1R7T362** | **Heat shock protein 83** | **3** | **1.8%** | **4.71** |
