# Supplementary material for: Crustacean Mab21 proteins drive tissue-specific antiviral immunity by activating IKKε outside the canonical nucleic-acid sensing paradigm
Source: PLoS Pathog. 2026 Feb 17;22(2):e1013986. doi: 10.1371/journal.ppat.1013986 (PMC12928593; doi:10.1371/journal.ppat.1013986)
Supplement: S3 Table — (DOCX) [file ppat.1013986.s007.docx]

**S3 Table****. Proteomic identification of candidates modified residues of LvIKKε based on LC-MS/MS analysis.** In “Variable modifications position”, “.” denotes an enzymatic cleavage site, “0” indicated no modification at this amino acid position, “1” indicated oxidation modification on methionine (M), “2” indicated phosphorylation modification on cysteine (C), “6” indicated phosphorylation modification on serine/threonine (S/T).

| **Peptide sequence** | **Variable modification** | **Variable modification positions** | **Peptide score** | **Peptide expect** |
| --- | --- | --- | --- | --- |
| **VGHVQSLTSAVSDR** | **Phospho (ST)** | **0.00000600000000.0** | **65.35** | **2.10E-05** |
| **FLMLTQMCGGNQESSSQPLR** | **2 Oxidation (M)** | **0.00100010000000000000.0** | **55.03** | **0.00053** |
| **FGSFPTLVSVEHDAAVGK** | **Phospho (ST)** | **0.006000000000000000.0** | **20.14** | **0.8** |
| **FLMLTQMCGGNQESSSQPLRER** | **Phospho (C)** | **0.0000000200000000000000.0** | **21.06** | **1.2** |
| **TPLVLFSK** | **Phospho (ST)** | **0.60000000.0** | **11.03** | **1.6** |
| **HLSDIVAPDQPSSSYPSTTPR** | **Phospho (ST)** | **0.006000000000000000000.0** | **16.82** | **2.5** |
| **TPLVLFSK** | **Phospho (ST)** | **0.00000060.0** | **6.1** | **3.9** |
| **KPVGKTFGAR** | **Phospho (ST)** | **0.0000060000.0** | **3.97** | **12** |
| **ASTYVTKLR** | **Phospho (ST)** | **0.060000000.0** | **2.88** | **18** |
| **SMCSVGHAIK** | **Phospho (ST)** | **0.6000000000.0** | **4.94** | **19** |
| **VGHVQSLTSAVSDR** | **Phospho (ST)** | **0.00000000000600.0** | **4.42** | **24** |
