# Supplementary material for: Crustacean Mab21 proteins drive tissue-specific antiviral immunity by activating IKKε outside the canonical nucleic-acid sensing paradigm
Source: PLoS Pathog. 2026 Feb 17;22(2):e1013986. doi: 10.1371/journal.ppat.1013986 (PMC12928593; doi:10.1371/journal.ppat.1013986)
Supplement: Attachment — Submitted filename: Response to reviewers.docx [file ppat.1013986.s010.docx]

**Additional Editor Comments:**

The Li et al., manuscript investigating three shrimp cGAS homolog Mab21 proteins has been reviewed by one reviewer in addition to the Editor. For reasons that remain unclear, it has been challenging to find reviewers willing to take on this story. In the manuscript, authors provide evidence supporting an interaction directly between Mab21 proteins and a shrimp TBK1 homolog of IKKε, promoting serine 175 phosphorylation and downstream IRF–Vago4 signaling that is tissue specific to each of the family members. Unlike vertebrate and insect counterparts, these proteins do not sense dsDNA/dsRNA or produce canonical cGAMP but are shown to act as protein-based enhancers of kinase activation.

Initially, the authors identify three Mab21 protein coding sequences in the genome of white Pacific shrimp but observe that all three lack consensus canonical nucleotidyltransferase domains characteristic of cGAS-like homologs that recognize either dsDNA or dsRNA in vertebrates and other invertebrates. Authors try and fail to see any evidence that the shrimp Mab21 proteins behave as nucleic acid sensors in conventional overexpression systems, negative experiments that are included in Fig 2 and discussed later in the manuscript. Authors proceed to coimmunoprecipitation of overexpressed proteins in HEK293T cell extracts revealed that all three Mab21 proteins interact with shrimp IKKε (a homolog of TBK1) and then proceed to characterize a critical phosphorylation site (S175) enhanced by any of the Mab21 proteins again by overexpression. The artificial circumstances where this work is carried out raises concerns that are not fully addressed as the data are generated and presented. These activities should be shown to occur in shrimp or shrimp cells concurrent with overexpression work. The work in shrimp systems in necessary to consider the Mab21 observations verified.

**Reply:** We thank you for this careful and constructive assessment and fully agree that validation of Mab21 function in shrimp systems is essential. Below, we clarify our experimental rationale and emphasize how our study integrates both heterologous and shrimp-based approaches to address this concern.

First, at present there is no established continuous cell line, nor a reliable *in vivo* overexpression system, available for *Litopenaeus vannamei*. Under these technical constraints, we employed a well-established heterologous system—MAVS-deficient HEK293T cells—to perform initial mechanistic analyses. This system has been widely used to dissect innate immune signaling pathways and allowed us to test whether shrimp Mab21 proteins could directly modulate IKKε activity in a controlled cellular context. Using this approach, we demonstrated that co-expression of LvMab21-1, LvMab21-2, or LvMab21-3 with shrimp IKKε consistently enhanced phosphorylation of IKKε at serine 175, a conserved activation site required for downstream IRF–Vago4 signaling.

Importantly, we did not rely solely on heterologous overexpression. To validate the physiological relevance of these findings in shrimp, we performed RNA interference–mediated knockdown of each Mab21 paralog in *L. vannamei*. We then examined endogenous IKKε phosphorylation, IRF activation, and Vago4 expression in multiple shrimp tissues, including hemocytes, hepatopancreas, gills, and intestine. These in vivo experiments demonstrated that depletion of LvMab21-1, LvMab21-2, or LvMab21-3 significantly reduced IKKε phosphorylation and downstream interferon-like signaling in a tissue-specific manner, fully consistent with our heterologous observations.

Thus, by combining mechanistic analyses in a heterologous system with tissue-resolved functional validation in shrimp, our study minimizes the limitations inherent to each individual approach. While we acknowledge that the absence of a shrimp overexpression cell system is a technical limitation of the field, the convergent evidence from both experimental platforms supports the conclusion that Mab21 proteins function as physiological regulators of IKKε-dependent antiviral immunity in shrimp.

Furthermore, given that this is about an interferon-like response, more needs to be done to show an antiviral impact akin to what is projected by authors comments. It is only in Fig. 5 where WSSV infection is shown to rapid and strong induction of LvMab21-1 ~10-fold by 36 h. This seems modest at best for interferon-type response both in induction level and kinetics and raises questions as to what signaling precedes the function of Mab21 proteins in the natural system. Nevertheless, RNAi knockdown of LvMab21-1, LvMab21-2, or LvMab21-3 is shown to significantly reduce phosphorylation of shrimp IKKε and activation of IRF and Vago4 in Fig. 6, consistent with the conclusion that “all three shrimp Mab21 proteins positively regulate the IKKε–IRF–Vago4 pathway in hemocytes”. Additional details are presented in Figs 7 and 8; however, there is too little attention to whether the pathway impedes WSSV infection dependent on the Mab21 proteins, all of which is fundamental to the authors inquiry. Besides the actual antiviral impact, the kinetics of the response should be expanded so as to understand whether this is an innate host antiviral response to virus infection that yields an active interferon.

**Reply:** Thank you for this important and insightful comment. We agree that demonstrating a clear antiviral impact and clarifying the kinetics of the Mab21-dependent interferon-like response are essential to support our conclusions. In response, we have expanded both our experimental evidence and the corresponding discussion in the revised manuscript.

First, we would like to clarify that interferon-like responses in crustaceans differ fundamentally from vertebrate type I interferon systems in both magnitude and kinetics. Unlike vertebrate IFNs, shrimp Vago proteins function as interferon-like cytokines that are typically induced with delayed kinetics and moderate fold changes following viral infection (Gao J, et al. *Cell reports*. 2021;36(13)), as reported previously for STING–IRF–Vago signaling during WSSV infection (Li H, et al. *The Journal of Immunology*. 2024;212(12):1945-57). Therefore, the observed ~10-fold induction of LvMab21-1 at 36 h post-infection is consistent with a regulated innate antiviral response in shrimp rather than an acute cytokine burst typical of vertebrate systems.

Second, to directly address whether Mab21 proteins functionally impede WSSV infection, we added and emphasized **new *in vivo* antiviral data (Figure 9)**. Using RNA interference to individually and combinedly silence LvMab21-1, LvMab21-2, or LvMab21-3, we assessed host survival and viral replication following WSSV challenge. In all knockdown groups, shrimp exhibited significantly increased mortality and markedly elevated viral genome copy numbers compared with controls. These results provide direct functional evidence that each Mab21 protein contributes to antiviral defense against WSSV.

Third, we examined the downstream interferon-like signaling cascade in the context of Mab21 depletion. Knockdown of LvMab21 proteins significantly reduced IKKε phosphorylation, IRF activation, and Vago4 expression in hemocytes and other immune-relevant tissues, establishing a mechanistic link between Mab21 activity and antiviral effector induction. Importantly, these effects were tissue-specific, indicating that Mab21 proteins act as modulators of localized innate immunity rather than global immune amplifiers.

Finally, regarding pathway kinetics and upstream regulation, our data suggest that Mab21 proteins do not serve as primary viral sensors but instead function downstream or parallel to pathogen recognition, acting as signal amplifiers or facilitators of IKKε activation once the interferon-like pathway is engaged. We have clarified this point in the revised Discussion to better distinguish Mab21 proteins from canonical nucleic acid sensors and to frame their role within the broader temporal architecture of shrimp innate immunity (lines 267-304).

**Journal Requirements:**

If the reviewer comments include a recommendation to cite specific previously published works, please review and evaluate these publications to determine whether they are relevant and should be cited. There is no requirement to cite these works unless the editor has indicated otherwise.

1) Please ensure that the CRediT author contributions listed for every co-author are completed accurately and in full.
At this stage, the following Authors/Authors require contributions: Chaozheng Li. Please ensure that the full contributions of each author are acknowledged in the "Add/Edit/Remove Authors" section of our submission form.
The list of CRediT author contributions may be found here:

<https://journals.plos.org/plospathogens/s/authorship#loc-author-contributions>

**Reply:** Thank you. The list of CRediT author contributions has been ensured.

2) We do not publish any copyright or trademark symbols that usually accompany proprietary names, eg ©, ®, or TM (e.g. next to drug or reagent names). Therefore, please remove all instances of trademark/copyright symbols throughout the text, including:
- TM on pages: 13, 15, and 16.

**Reply:** Thank you. All instances of trademark/copyright symbols have been removed.

3) Please upload all main figures as separate Figure files in .tif or .eps format. For more information about how to convert and format your figure files please see our guidelines: 
<https://journals.plos.org/plospathogens/s/figures>

**Reply:** Thank you. All main figures have been uploaded.

4) Some material included in your submission may be copyrighted. According to PLOSu2019s copyright policy, authors who use figures or other material (e.g., graphics, clipart, maps) from another author or copyright holder must demonstrate or obtain permission to publish this material under the Creative Commons Attribution 4.0 International (CC BY 4.0) License used by PLOS journals. Please closely review the details of PLOSu2019s copyright requirements here: PLOS Licenses and Copyright. If you need to request permissions from a copyright holder, you may use PLOS's Copyright Content Permission form.
Please respond directly to this email and provide any known details concerning your material's license terms and permissions required for reuse, even if you have not yet obtained copyright permissions or are unsure of your material's copyright compatibility. Once you have responded and addressed all other outstanding technical requirements, you may resubmit your manuscript within Editorial Manager.

Potential Copyright Issues:

i) Please confirm (a) that you are the photographer of 9C, or (b) provide written permission from the photographer to publish the photo(s) under our CC BY 4.0 license.

ii) Figures 1C, and 6A. Please confirm whether you drew the images / clip-art within the figure panels by hand. If you did not draw the images, please provide (a) a link to the source of the images or icons and their license / terms of use; or (b) written permission from the copyright holder to publish the images or icons under our CC BY 4.0 license. Alternatively, you may replace the images with open source alternatives. See these open source resources you may use to replace images / clip-art:

**https://commons.wikimedia.orgReply:** Thank you for your careful review regarding image copyright. We respond to your questions point by point as follows:

(1) The silhouette icons in Figure 1C

We have deleted the silhouette icons in Figure 1C.

(2) The images in Figure 6A

The schematic diagram in this figure is an original illustration hand-drawn by our team specifically for this manuscript. The copyright belongs to the authors, and it can be published directly under the CC BY 4.0 license.

(3) The photograph in Figure 9C (now Figure 9D after revision)

This photograph was taken by a member of our research team during the experiments. The copyright belongs to the authors, and it meets the requirements for publication under the CC BY 4.0 license.

We confirm that all the above images are free of third-party copyright issues, and we are prepared to provide relevant statements or authorization documents as required by the journal.

**Reviewers' Comments:**

Reviewer's Responses to Questions

**Part I - Summary**
Please use this section to discuss strengths/weaknesses of study, novelty/significance, general execution and scholarship.

**Reviewer #1:** In this new submission, teh authors investigate the role of cGAS like Mab21 proteins in the shrimp antiviral immune response. Surprising they fail to identify either nucleic acid binding activity nor CDN synthesizing activity. Instead, they identify interactions with the shrimp Mab21 proteins (3 ISSO forms) and the IKKe homolog, leading to IKK phosphorylation/activation. They map an activating phosphorylation site in shrimp IKKe, at a location that is orhtologous to mammalian IKKe; presumably this is an autophosphoyrlation event, although that is not demonstrated.

Using RNAi approaches in shrimp, they show these Mab21 proteins are critical for IKKe phosphorylation/activation as well as IRF dimerization, with varying levels of expression/inducaton and relevance in different shrimp tissues.

**Part II – Major Issues: Key Experiments Required for Acceptance**
**Reviewer #1:**

**1.** The authors should address, more directly, if the Mab21 have a charged surface, like cGAS and cGLRs, that bind nucleic acid. They also mention, but only in the Discussion, the shrimp Mab21s lack catalytic residues this should all be directly demonstrated and displayed in the Results and Figure 1.

**Reply:** Thank you for this valuable suggestion. We have now directly addressed both points by incorporating additional analyses into the Results section and revising Figure 1 accordingly, as detailed below.

**(1) Charged surface features relevant to nucleic acid binding**

To determine whether shrimp Mab21 proteins possess surface properties similar to those of cGAS/cGLRs that mediate nucleic acid binding, we explicitly annotated key positively charged residues involved in nucleic acid recognition in human cGAS and compared them with the corresponding regions in *Litopenaeus vannamei* Mab21 proteins. These residues are now highlighted in the multiple sequence alignment presented in Figure 1A.

In addition, we constructed three-dimensional structural models of the Mab21 domains and analyzed their electrostatic surface potential using Discovery Studio (Figure 1C). This analysis revealed the presence of a surface groove enriched in positively charged residues, suggesting a potential interface for ligand or protein interaction. These results and their implications are now described in the Results section (lines 129–132 and 146–147).

**(2) Absence of conserved catalytic residues**

To directly demonstrate that shrimp Mab21 proteins lack catalytic capacity, we have incorporated this analysis into the Results section and Figure 1. In the revised multiple sequence alignment (Figure 1A), residues essential for nucleotidyltransferase activity in canonical cGAS/cGLRs are clearly marked. This comparison shows that LvMab21-1, LvMab21-2, and LvMab21-3 lack these conserved catalytic residues, providing direct sequence-based evidence that they do not possess canonical enzymatic activity required for cyclic dinucleotide synthesis.

Together, these revisions ensure that both the surface charge characteristics and the lack of conserved catalytic residues in shrimp Mab21 proteins are explicitly demonstrated in the Results and Figure 1, rather than being discussed only conceptually.

**2.** Throughout the manuscript, when more than two groups are compared, an ANOVA type analysis should be used to analyze for significance (t-test not sufficient).

**Reply:** Thank you for this important comment. In response to this suggestion, we have re-analyzed all datasets involving comparisons among more than two groups throughout the manuscript. Specifically, we have replaced the previously used Student’s *t*-tests with one-way ANOVA and updated the corresponding statistical analyses, figure annotations, and result presentations in Figures 2B–C, 3B, 4A, 6B, 6D, and 8A.

**3.** The effects of Mab21 silencing in hemocytes is modest (6C), at best. This and other key immunoblots should be included replicates and quantitation. The data from the hepatopancreas is much more robust. The blots from the intestines - not convincing. These blots are critical linchpins for this story and all should be more convincing and replicates shown and quantified, to demonstrate rigor and reproducibility.

**Reply:** We thank the reviewer for this important and constructive comment. In response, we have supplemented and strengthened the key immunoblot data to improve rigor, reproducibility, and quantitative support, as detailed below.

**(1)** These blots were further subjected to densitometric analysis, including quantification of IRF dimer-to-monomer ratios and levels of phosphorylated IKKε normalized to Actin.

**(2)** In addition, we repeated the immunoblot experiments using independent biological samples from hemocytes, hepatopancreas, gill, and intestine. The repeated immunoblot results, together with their corresponding quantitative analyses, have been incorporated into the revised manuscript and are presented in Figures 6C and 8B, accompanied by densitometric quantification.

These additions provide quantitative validation of the observed effects across tissues and address concerns regarding variability and reproducibility of the immunoblot data.

4. The sensitivity to infection with individual RNAi targeting of each Mab21 is also modest. The authors should knockdown multiple (or all 3) Mab21s, as there model predicts (at least additive) effects of knocking down each, with hypothesized roles in distinct tissues.

**Reply:** Thank you for this important and insightful suggestion. In response, we have performed additional knockdown experiments to examine whether Mab21 proteins exert additive or cooperative antiviral effects across tissues, as predicted by our proposed model.

Specifically, we repeated the WSSV infection challenge experiments using not only individual knockdown of LvMab21-1, LvMab21-2, and LvMab21-3, but also combined knockdown of all three genes. We then systematically compared shrimp mortality rates and viral genome copy numbers among these groups.

The results showed that triple knockdown of LvMab21-1/2/3 resulted in more pronounced increases in mortality and significantly enhanced viral replication compared with individual knockdowns. These findings support the hypothesis that Mab21 proteins function in a cooperative or additive manner to regulate antiviral immunity across distinct tissues.

The updated survival curves and quantitative viral load data have been incorporated into Figure 9, and the corresponding descriptions have been added to the revised manuscript (lines 247–258).

**Part III – Minor Issues: Editorial and Data Presentation Modifications**
**Reviewer #1:**

1. Suggested to reorganize data in Figure 7, so the changes with time/infection/challenge and distinction between tissues is more easily comprehended.

**Reply:** Thank you for this helpful suggestion. To improve clarity and data presentation, we have reorganized Figure 7 based on the different immune stimuli. Specifically, the original Figure 7 has been divided into a revised main Figure 7 and Supplementary Figure S3, with each figure presenting results from separate experimental conditions.

In addition, the original bar charts have been replaced with line charts to more clearly visualize temporal dynamics of LvMab21 expression in each tissue and to facilitate comparison of response kinetics across tissues. These changes improve readability and better convey tissue-specific and time-dependent expression patterns.

2. The first paragraph of the Discussion should be reworded to be NOT over interpret the negative conclusions regarding sensing and CDN synthesis. The following paragraph does an excellent job explaining the caveats, but the first paragraph overstates the conclusions.

**Reply:** Thank you. In response, we have revised the first paragraph of the Discussion to present our negative findings regarding nucleic acid sensing and CDN synthesis more cautiously. The revised text now explicitly states that these conclusions are based on the specific experimental conditions employed and avoids implying a complete absence of nucleic acid recognition or signaling capacity.

Furthermore, we have added a transitional statement at the end of the first paragraph to better connect it with the subsequent discussion, which addresses potential limitations and alternative possibilities. This revision ensures a more balanced interpretation and improves the overall logical flow of the Discussion.
